# Property as the law of virtual things

**DOI:** 10.3389/frma.2022.981964

**Published:** 2022-08-26

**Authors:** Joshua Fairfield

**Affiliations:** School of Law, Washington and Lee University, Lexington, KY, United States

**Keywords:** property, NFT, non-fungible token, virtual, scarcity and abundance, law

## Abstract

Property law in the twentieth century moved from the law of things to the law of rights in things. This was a process of fragmentation: Under Hohfeldian property, we conceive of property as a bundle of sticks, and those sticks can be moved to different holders; the right to possess can be separated from the record ownership right, for example. The downside of Hohfeld's model is that physical objects—things—become informationally complicated. Thing-ness constrains the extravagances of Hohfeldian property: although we can split off the right to possess from the right to exclude, use, destroy, copy, manage, repair, and so on, there is a gravitational pull to tie these sticks back into a useful bundle centered on the asset, the thing. Correspondingly, there has been an “informational turn” to property law, looking at the ways in which property law serves to limit property forms to reduce search costs, and to identify and celebrate the informational characteristics of thing-ness. The question of thing-ness came to a head in the context of digital and smart assets with the formation of non-fungible tokens. NFTs were attempts to generate and sell “things,” a conceptually coherent something that can contain a loose bundle of rights. The project was an attempt to re-create thing-ness by an amalgam of cryptography, game theory, and intellectual property. This essay discusses thing-ness in the context of digital assets, how simulated thing-ness differs from physical thing-ness, and the problems that arise from attempts to reify digital assets.

## Introduction

Property law in the twentieth century moved from the law of things to the law of rights in things. This was a process of fragmentation: Under Hohfeldian property, we conceive of property as a bundle of sticks, and those sticks can be moved to different holders; the right to possess can be separated from the record ownership right, for example. The downside of Hohfeld's model is that physical objects–things–become informationally complicated. A simple farm can have complex arrangements of owners, easements, and servitudes. Things no longer contain and constrain complexity within themselves.

Thing-ness constrains the extravagances of Hohfeldian property: although we can split off the right to possess from the right to exclude, use, destroy, copy, manage, repair, and so on, there is a gravitational pull to tie these sticks back into a useful bundle centered on the asset, the thing. Conceptions of thing-ness helps with this process by conveying information quickly and easily (the person wearing the watch is probably its owner), by providing smooth and modular interfaces that contain complexity (think about the complexity of a car engine constrained within the thing-ness of the car, or of the complexities of circuits contained within a laptop, or the like), and reduce the number of property forms so that people searching for property do not incur large search costs due to uncertainty about what they will buy. Correspondingly, Henry Smith, Tom Merrill, Christina Mulligan, I myself, and others have taken what I term an “informational turn” to property law, looking at the ways in which property law serves to limit property forms to reduce search costs, and to identify and celebrate the informational characteristics of thing-ness.

The question of thing-ness came to a head in the context of digital and smart assets with the formation of Non-fungible tokens. NFTs were attempts to generate and sell “things,” a conceptually coherent *something* that can contain a loose bundle of rights. The project was an attempt to re-create thing-ness by an amalgam of cryptography, game theory, and intellectual property. An NFT is a loosely bundled mixture of a cryptographic token often hyperlinked (or otherwise loosely associated) with a piece of intellectual property–a jpeg, for example. The social description of an NFT as a thing gives the NFT, the amalgam, a conceptual box that bounds what is bought and sold. The resulting loose associations have had enormous success as rivalrous, scarce, valuable digital “things” in communities of collectors who are enamored of the uniqueness component offered by the digital ledger, and the sense of scarcity it imparts to what are otherwise standard easily copyable computer files. They have also suffered enormous setbacks because of the same issues. NFTs are mulcted as being “nothing,” and thus worth nothing, when the thing-ness process fails.

The question is how “solid” the thing-ness of NFTs or other intangible personal property rights can be, how successful their socio-technological thingification has been. They are certainly solid enough to cause buyers to pay $69 million for a jpeg associated with a cryptographic token, or hundreds of thousands of dollars for a short clip associated with a slot on a decentralized ledger. But for these assets to hold value (and in the current meltdown we must ask whether they will succeed) we must be able to look at what they are–their thing-ness–and determine whether the conceptual container of a digital thing is strong enough to hold the legal rights.

Hohfeldian property was a process of adding informational characteristics to real and personal property. The development of NFTs involves adding technologically created physical characteristics to informational objects. Thing-ness is needed as a constraint to complexity, as a force for defragmentation, and as a mold for modularity to help counterbalance the nature of digital objects and their tendency to fragment and dissipate, just as Hohfeldian legal rights were needed to add flexibility and free up value in real and personal property. This essay discusses thing-ness in the context of digital assets, how simulated thing-ness differs from physical thing-ness, and the informational problems that arise from attempts to reify digital assets. It thus attempts to do two things at once: to discuss what information-based property theory can say about the attempt to create digital things, and what the strong and clear example of NFTs can do to forward and develop property theory.

## The history of property online

Technological shifts spur legal shifts. As internet technologies developed, legal norms shifted rapidly. Some areas managed the shift to digital technology relatively seamlessly. Contract shifted to electronic contract with a minimum of fuss.^1^ To be sure, the shift in affordances worked deep changes that have changed the face of contract law forever. Electronic contracts eventually changed the nature of contracting from dickered mutual agreement to the EULA.^2^ But at no point did the new technology prevent contracts from forming even if the contract took a different form and protected different interests.

Not so with property. Unlike contracting online, which was able to survive for the most part, parties have, until now, not been able to create robust electronic personal property interests. The dominant paradigm for property online became intellectual property.^3^ The fit was not quite right. Intellectual property did indeed deal with intangibles, but regular rights in property—easements, possibilities of reverter, a renter's right to present possession of a rental car, and so on—are quite intangible too. The shift online knocked out almost all rights in personal property, and replaced them with intellectual property licenses.^4^ We do not own our fully paid-for eBooks, movies, games, and so on, we merely license them.^5^

Property law organizes peoples' rights with respect to scarce resources. It is the word *scarce* that interests us here. Consider a book. There are personal property interests in the physical copy, and intellectual property interests in the copyrightable writing. The book can be kept scarce by limiting the number of physical objects made. Intellectual property interests limit the ability of free riders to simply make infinite copies of the book. This changed online—the limitation of the physical form gone, anyone could make infinite copies of a work at near zero cost. The assumption of law was that the physical form of the *copy* was gone, and all that remained was the copyright.^6^ The law sought to recreate scarcity by imposing sanctions on anyone who made a copy.^7^ The approach had the unfortunate side effect of eradicating traditional personal property interests. That personal property interests are present online is clear, but only when intellectual property rights do not muddy the question. Thus, for example, a domain name is considered personal property.^8^ As *Kremen v. Cohen* noted, property extends to anything susceptible to unique possession.^9^ Yet until relatively recently, intellectual property interests and ubiquitous End User License Agreements obscured nearly all cases of digital personal property.^10^

These twin problems of IP overreach and lack of true digital scarcity so plagued online personal property interests that they did not make a robust transition to online environments. The result is now over two decades of studies showing that digital items and online assets are worth billions of dollars, yet all of these markets are at best gray because law has failed to offer a coherent framework for digital ownership.^11^

### Digital scarcity and value

The mismatch between consumer expectations for ownership of digital property and what has been made available in the form of EULA-enabled use of digital property is the result of decisions—both legal and technological—made during the early days of the internet. The early fears of digital property are encapsulated in the Napster story.^12^ Napster enabled individual users to share music files at near-zero cost and skirt IP protections.^13^ The response was to bolster protections for copyrighted material, but this is only as effective as the capability of its enforcement. It's too difficult to enforce the protections against every person with a computer.

The answer was technologically enforced digital scarcity. Developers placed a series of locks on users' personal devices that are collectively referred to as Digital Rights Management or DRM.^14^ DRM programs can prevent you from downloading a DVD to your computer or converting a YouTube song into an MP3. As anyone who has ever thought about downloading a YouTube video knows, those protections can often be defeated with a simple Google search. Whenever a new DRM control is created, people with technological expertise set out trying to defeat it.^15^ Rather than engaging in a DRM arms race, those trying to protect copyrighted material lobbied Congress to make it illegal to break the DRM locks on devices and to help others break those locks.^16^ Now, rather than chasing down every person who has ever converted a YouTube video to an MP3, companies only need to go after the people making YouTube to MP3 conversion programs. The average person does not have the technological know-how to break a DRM lock on their own, so by preventing people from creating the means of breaking DRM locks, copyright protectors thought (incorrectly as it turned out) that they had found the key they needed to artificially create digital scarcity and protect value online. The first problem was that DRM proved simply too easy to skirt. The second was that this means of creating digital scarcity could only be accessed by a small group of people—large corporations with thousands of copyrights and billions of dollars to create DRM controls over their products as they circulate on the internet. A means of protecting scarcity (and thus value) that is only available to one group of people means that the value created by those items is only available to one group of people as well.

The centralized license server DRM model succeeded only in imposing constraints on consumers and owners, not pirates. Online assets are therefore in the first stages of migrating from the failed traditional centralized command-and-control model to a decentralized model of individual ownership. The technological change that undergirds this shift is the development of blockchain technology, a form of decentralized database that merges encryption and game theory to create lists of ownership that do not rely on any central entity to maintain the list, and are robust against efforts to falsify the lists.^17^ The social shift is one in which large numbers of people have created a social context for value in digital property.

### Value is social

The value of a thing of course does not reside in that thing itself, but in the value that social groups attach to it. As a social group values or demands a thing more, its price rises; this is the basic mechanism of scarcity and value. There are different components: a thing must be desirable in some way, and scarcity exacerbates demand. Increasing value in the face of scarcity is often less of a mystery than value in the first place. We understand demand for gasoline, and how the value can rise as supply becomes scarce more than how a new form of demand comes into being. Consider the kind of demand sufficient to support paying thousands of dollars for a GIF of a fun play in an NBA game, for example.^18^ It is important not to spend too much time asking why people value it (one useful exercise for the reader may be to examine their own hobbies and ask what they might pay for an object with particular value within that activity, in order to see how communities generate value), and instead focus on the mechanisms by which law reduces transaction costs for satisfying human preferences.

Social value has two components, a community that attaches value and a nexus to which community value attaches. Social value alone is not enough: imagine that a community of sports fans attaches particular importance to a moment in sports history. That is a shared experience, non-rivalrous, a potential source of value, but without a mechanism to attach that value. Tying the moment to an entry in a cryptoledger, and creating a community that recognizes the owner of the ledger entry as having some special relationship with the moment takes turning an experience into a thing.

As an aside, it is worth asking whether privatizing a moment by creating a collectible is a socially beneficial activity. Why take something shared and create something that can be owned by an individual? Yet it is not clear that the existence of band merchandise reduces the social value of a concert, that a home-run ball reduces the social value of a baseball game, or that a community of collectors of artifacts from the Alamo reduces the social significance of the historical moment. Things can serve as a way of helping those who value an experience to convey value to the community of interest. Buying artwork is a core way of supporting art, and so on.

Things are commonly accepted means for turning social value into a collectible: consider for example a homerun baseball or the tickets to a culturally significant concert. Anyone who has been a collector or watched a collectors' market has seen how mundane objects gain value by association with a socially relevant moment. To carry this freight, things must be *authentic*. As will be discussed further down, blockchains, NFTs, and cryptoledger technology solve the problems of how to attach value and authenticate objects, items, and experiences. Addressing these problems allows for social value to be stored and owned in the digital space in the same way that it is in the physical space.

## Property and information theory in the law of things

This sub-part considers the future of the tension between Hohfeldian property theory, and the subsequent turn to information theory, which seeks to limit the extent to which Hohfeldian disaggregation of property rights raises transaction costs.

### Hohfeldian property and information theory

Hohfeldian conceptions of property have dominated our understanding of property interests long before the law began adapting itself to new digital contexts. Conceptualizing property as a bundle of sticks helped jurists, scholars, lawyers, and average users of property understand how different interests can be bought or sold. It freed legal rights from the constraints of thing-ness: I can own something even though you possess and use it, and so on. The Hohfeldian conception of property freed up property use, but without the boundaries of thing-ness it can become self-defeating. Property can become fragmented to the point of uselessness when too many sticks are siphoned away from the bundle, when ownership is too divided, or when interests are too informationally complex for buyers to know what they are getting. Information theory seeks to tie the interests back together and re-create *thing*-ness to prevent property from being fragmented to the point of uselessness.^19^

A group of theorists and theories (Henry Smith, Tom Merrill, Christina Mulligan, myself, and others) take what I term an “informational turn.” Under this view, thing-ness in property law limits fragmentation of property rights, exerts a gravitational pull on titles that are more marketable, and works to eliminate invisible interests that raise search costs.^20^ While property enables fractional and divided ownership by, say, allowing parties to move sticks like servitudes, easements, and the like from one holder to another, there are any number of doctrines that reinforce that the contours of the legal right should match the contours of the thing, and limit rights to the extent that they complicate the use or marketability of the thing, whether the thing be a diamond, a farm, a factory, or an NFT.

Property is the discipline of determining rights between humans with respect to scarce resources. The law is primarily concerned with conveying information about who may do what with which resources. A primary goal is ensuring that the rights pass smoothly in the stream of commerce. The most important person in property law is the uninvolved third party—an interested buyer, seller, potential trespasser, or the like—someone who does not know the lay of the land, does not know about any hidden deals made between prior owners of the land and any other parties.

The problem is that Hohfeldian sticks, when removed from the bundle, complicate the informational characteristics of the property. To enable Hohfeldian disaggregation of the bundle of sticks, to enable things like easements, databases become necessary technology. Expensive and often inaccurate title searches are necessary because, with property, what you see is not what you get. The use characteristics of the property are not immediately available to third parties who may wish to purchase, rent, or even simply take a hike on the property.

### The numerus clausus and search costs

To respond to needs created by Hohfeldian property interests, information theorists have identified at least four ways in which property law acts to limit the impact of splitting up rights in things, especially when splitting up rights in things impedes the free flow of the asset in the stream of commerce or impedes the use of the thing. The first is the idea of the *numerus clausus*, a civil law term that describes property law's reluctance to countenance new forms of ownership.^21^ Closing the number of property forms limits the range of information costs. Since Hohfeldian property rights are invisible on the face of the property, the theory goes, they must be kept few in number, clearly described, and must be written in the database in order to be enforced against third parties who otherwise have no way of knowing from the face of the property what sticks have been taken out of the bundle of fee simple ownership.^22^

The problem that Merrill, Smith, and others have proposed is that each variation from the default form of fee simple ownership increases search costs.^23^ The basic concept is easy enough to see: imagine that the only form of property ownership is fee simple absolute. One would not have to incur search costs in order to find out what rights came with the property. The answer would be simple: all sticks in the bundle reside with the owner. Indeed, in that scenario there would be no need for the bundle of sticks metaphor, because property ownership would not be decomposable.

There is an informational price to be paid for bespoke property forms. Everyone who has purchased a house has felt this cost, as they have either paid for it in money—by procuring a title search or paying for title insurance—or in time, as they have pored over plats attempting to understand easements and servitudes and the impact they have on the property. And to keep our eye on the ball: that search cost is particularly high in the sale of high-value intangible personal property interests, where a code audit of the smart contract and legal analysis of intellectual property licensing agreements will be at a bare minimum necessary to determine what exactly an investor or collector has bought.

Merrill and Smith offer the central example of a bicycle. What if we could sell off (not contract out of, but actually sell the Hohfeldian stick out of the bundle) the right to use the bicycle on Tuesday mornings?^24^ If that were the case, the legal damage done by the prying of a stick out of the bundle of rights would not be apparent on the surface of the bicycle, and yet the damage would certainly be done. The bicycle would be worth less with the right sold off, but that is the lesser problem. If people have the ability to sell off such rights in their bicycles, *all* bicycles would cost more to acquire, since prospective owners must now search for and ensure that they do not run afoul of a right that has already been sold off.

Merrill and Smith's key example is drawn from personal property, and with good reason—we do not have formal methods for owning personal property with easements and servitudes. Fee simple absolute is in fact the norm for personal property, and possession is usually deemed synonymous with ownership: there is (rather, there was) no record of personal property ownership because there didn't need to be.

Particularly in the realm of personal property, *thing-ness* and the possession of things carry enormous informational freight. Possession of a thing conveys information because the thing is rivalrous, because it is an integrated whole, the wheels come with the car, the right to use the car comes with it as well. A thing in the law of property is what Latour calls a quasi-object. Like a brick (given its shape by physics and culture), a thing is an amalgam of understandings about the extent of resources conveyed with the thing, the rights conveyed with the thing, a mixture of material affordance and social permission.^25^ Consider the act of buying a washing machine at a yard sale. There is no record of ownership, there is no fragmentation of the rights in the machine, one expects to simply buy the machine and have all rights in it, to be done with the matter. The all-important information conveyed to the buyer is that they may buy a set of resources and rights, all packaged modularly, to flow in the stream of commerce, in the form of ownership of the thing. Thing-ness carries all of that information in the webwork of understanding humans have worked out with each other.^26^

### Defragmentation

Thing-ness also addresses the Hohfeldian problem of fragmentation by *defragmenting* property so that it is compiled into a single *thing*. Imagine a tractor. It would make little sense to carve up ownership of a tractor in such a way that one person would own the steering wheel, another the engine, the third the wheels. For a more concrete analogy, consider the difficulty of land that, through descent and distribution, comes to be owned by many people. The use of such land becomes complicated. The law assumes that co-tenants each have total rights over co-owned property, but the practicality is that land subject to fragmented property interests is worth less, is harder to sell, and is harder to use, because of the multiplicity of overlapping interests.^27^

The point of property is the ownership and use of *something*. Thing-ness, the idea that the bundle of rights relates to some core conceptual object, returns Hohfeldian sticks to the original bundle unless stringent formalities of notice are met. It provides an out, restoring co-owned property to single title ownership through partition by sale, and so on.^28^ Put another way, the law has a series of built-in systems that continually work to align the Hohfeldian interests with actual asset. To provide just one example, consider how the law of adverse possession aligns record title ownership with the actual on-the-ground use and possession of property.

Property is information, whether written in a ledger or written on the landscape.^29^ Where ownership interests diverge from what is plainly visible, databases fill the gap. Where the database written on the landscape diverge from the informational databases, we reconcile the two.^30^ Given, then, that property is so heavily involved with information, it is perhaps superficially surprising that its transition to fully informational (i.e., virtual) environments has been so fraught.

### Modularity

A third component of thing-ness with respect to information is modularity. Consider a car muffler. The capabilities of the muffler could have been engineered into the car itself.^31^ (The opposite is often also true: status “performance” vehicles and electric cars sometimes have noise generators so that people can hear the car coming.) The point, though, is that certain components, like mufflers, oil filters, alternators, and so on, are designed to be modular, to be easily swappable.

Thing-ness in this respect is a matter of constraining the inputs and outputs of a module. Modules contain complexity. The inside of a swappable component can be as complex as one wants, as long as the interface with the rest of the system is managed by a simple plug. If anyone has installed RAM into a computer, they get the point. The RAM sticks are the result of tremendous innovation in the number of circuits contained in a chip, they are absurdly internally complex. That complexity needs to be swappable, however, and so the thing, the stick of RAM, has a clean plug that allows it to interface with the rest of the system. Thing-ness helps systems become interoperable and interchangeable. The thing is the unit of complexity that is low-cost to swap.^32^ Thing-ness in this regard makes systems marketable, dis-assemblable, repairable, and upgradeable.^33^ When one thing can be swapped out without compromising other elements or parts of a system, it creates not only a market for the sub-level things themselves, but improves the value of things comprised of other things. Consider the market for cars, where the availability of parts and ease of repairability are significant components to the value of the car. A car that cannot be repaired, for which repairs involve work on complex, interconnected systems, lowers the value of the whole. Cars with widely available, easily swappable components are easier to repair and easier to upgrade. There are developed and competitive markets for the parts, which are each swappable. Making something integrated is a means of preventing competition on that component—consider how hard Microsoft labored to leverage its computer operating system monopoly into a monopoly on browser content: an attempt that failed despite Microsoft's integration of its inferior Explorer browser into every operating system because of the inherent modularity of software. Other options, Chrome, Firefox, etc., were easy to download and install. The worst features of software are often made integral, impossible to lever out, while superior products are made to be modular, useful in a wide range of contexts without compromising the surrounding systems or the integrity of the whole.

### Excludability and rivalrousness

The key feature of physical personal property is that it is excludable. If I have the ball, you do not. If I throw you the ball, you have it and I do not. Such an asset may also be rivalrous: if I consume it, that may reduce the amount of it there is for you to consume. Excludability has a strange half-defined relationship with scarcity. If there is a scarcity of balls, the physical excludability of the ball matters. If it does not, then excludability or rivalry may exist, but do not matter. Excludability and rivalry drive related concepts of uniqueness—an idea cannot be unique, since everyone can share it, and consuming it does not reduce the amount of the idea available to others—and scarcity, which have two significant inputs into the production and sale of excludable or rivalrous personal property.^34^ First, if an asset is not excludable or rivalrous, the marginal cost of production is usually quite low—it takes little to no cost to duplicate an idea or an MP3.^35^ This is Posner's well-established differentiation between personal and intellectual property. Personal property costs more or less the same for each marginal unit produced (with economies of scale, to be sure). The second is that the price for an excludable or rivalrous item reflects its (relative, often manufactured) scarcity. If an asset is truly non-excludable or non-rival and there are no effective access controls, then no-one will pay for it: it is available for free. An example might be—under normal circumstances—air and oxygen. But, like water (consider Evian or Fiji) assets may become valuable if they become scarce or artificial scarcity is imposed by imposing effective access controls.

Natural, physical excludability is the way the physical characteristics of things became informational: things, because of the natural costs of making more of them, carry the freight of the system of value by which creators (and unfortunately middlemen) are compensated. As each person buys a record, a copy of a book, a CD or copy of a movie, the creator is remitted royalties, for example. If copies are free—as in rampant digital piracy—then this value chain breaks down. The value of the goods is zero if they are truly non-excludable or non-rival: the Nash equilibrium for price goes to zero when creators are forced to compete against entities that can provide copies at zero cost.

As noted elsewhere, intellectual property extended and evolved to increase access controls, both to create the artificial scarcity needed to produce the kind of value delivery system thing-ness naturally provides. This attempt to create artificial scarcity by defending technological locks by law revealed its own Achilles heel: technological protection measures that rely on the protection of law are not much in the way of technological protection measures. Indeed, the history of such measures has been one of abysmal failure—copy protection measures are circumvented by hackers within weeks of being deployed. The lasting legacy of this ill-fated arms race between technological protection measures and hackers was only to increase IP rightsholders' control over users' rights beyond any consideration of the copyright.

All of which to say, technological means to create rivalry, scarcity, and uniqueness have been crucial goals of digital markets.

## Re-creating thing-ness in NFTs

Where Hohfeldian property conceptions worked to attach informational characteristics to physical property, the task for NFTs is to create simulated physical characteristics (excludability or rivalry chief among them) for pieces of property that are entirely comprised of information. Thing-ness, as it was useful in constraining informational complexity in property, will be equally useful in attempting to bond together the diffuse interests related to digital ownership.

Beginning with excludability, distributed ledgers—blockchains and the like—have sought to recreate certain characteristics of “thing-ness.” This allows the creators of these objects to tap into the intuitions around property, the set of widely installed social instructions that says that you are allowed to ride a bicycle you purchase, but not through someone else's living room. It allows sellers to capture the value associated with ownership, that loosely negotiated but highly prized set of social permissions around the use of scarce resources.^36^ With “thing-ness,” creators can get an item's sale price rather than its rental price. They can tap into the premium paid by people who want control of and access to a resource without interference from others, or those who wish to use ownership as an associational channel—if I own the Hope Diamond, I have acquired a certain *je ne sais quoi*.

As informational objects, NFTs of course can best be understood by attending to informational flows and forms. As I detail below, however, the informational characteristics of things are imperfectly recreated when the resources in play are non-physical. I do not mean that the features of thing-ness cannot be recreated. Much of what makes a thing a thing in property is the conveyance of rights that are a function of social agreement, not physics. NFT creators have invoked powerful intuitions around thing-ness and ownership. Yet they do not presently deliver on those features. This is because the legal framework that underlies NFTs details a different set of social expectations and affordances than one receives as the owner of personal property.^37^ Many NFT owners are surprised to learn how little they truly own. The following sub-parts pick apart our attempts to re-create thing-ness in information environments, and apply the information theory of property to the resulting digital quasi-objects, to see how they stack up.

## Nature of the non-fungible thing

A full treatment of cryptoledgers and cryptocurrency is beyond the scope of this short essay. For purposes of the discussion of how property—and particularly information theories of property—might serve as the law of virtual things, a few basic points are worth stressing.

The major problem for digital property was excludability—how to solve the online zero-cost copying problem. As noted above, the key distinction lies in marginal costs of production. A physical house costs as much to build the second time as it did the first. A virtual home is duplicated at the click of a button. Given that virtual assets are—without more—often duplicatable for near-zero cost, the law of intellectual property was given free rein online. For example, the ability to infinitely duplicate movies and music at no cost—a basic feature of the internet—was treated as an existential challenge by various industry associations who profited from artists and consumers alike by dominating distribution channels. Under their lobbying and control, intellectual property licenses created the current landscape, where owners merely license rather than own even fully paid-for digital assets. This copying problem ensured that copy-rights became the dominant legal regime, as industry-sponsored laws strengthened and extended license rights and increased penalties for helping owners make full use of their own purchases.

Copying was the same core problem in the attempt to solve a slightly different problem, that of creating a fully decentralized digital currency. Centralized currencies were not particularly difficult, requiring only a trusted entity to maintain a ledger and authenticate transactions. That raised two problems in turn: first, that the authenticator might not be trustworthy, or second, that the central ledger might be compromised by bad actors. In either case, the problem became the same as that of the intellectual property rights organizations: copying. The risk was that a bad actor might duplicate currency, commonly called the “double spending problem.” An actor might spend money, then rewrite the ledger, and spend it again, a modern version of check fraud by bouncing checks.

The solution was a combination of cryptography and game theory. Mathematical relationships tied entries into a database to one another, such that altering the past would alter the present—everyone would know that the database had been faked. Making that database and those linked entries is costly in terms of processing power (and energy, which makes the technology wildly damaging to the environment). The game theory component consisted of the fact that the only way to fake the database (and thus double-spend by rewriting the database to indicate that the spender had their money back) was to expend so many resources that it would be far more profitable to contribute to the main database than to hack it.

The result was a database of linked entries. If Person A sent Bitcoin 1 to Person B, and then later attempted to rewrite the common database to claw the bitcoin back, the effort would either prove futile, or, if enough processor cycles and energy were expended to functionally recreate the database, the database would come into question, destroying the value of all entries and therefore denying the fraudster of their prize.

The resulting digital assets were therefore excludable, and if consumed, rivalrous. If Person A transferred a bitcoin to Person B, the decentralized cryptoledger would register the transfer, and rewriting the history of the transaction was not feasible. In this way, the ledger digitally mimicked the excludable characteristic of physical personal property. The tokens were, however, largely fungible. Each bitcoin—or ether, or dogecoin, or whatever the cryptocurrency happened to be—was worth as much as any other. They were like quarters—scarce, valuable, but each much like any other, interchangeable.

Yet the analogy to quarters holds in one other respect. Some coins are collectible because of other facts or attributes, years, materials, history, and the like. They take on the characteristics of uniqueness. Even among bitcoin, these secondary characteristics offered a kind of differentiation if not uniqueness. For example, since every transaction of a coin is recorded as a transfer from one account to another recorded in a decentralized ledger, the entire transactional history of a unit is a matter of record. So, drug dealers prefer newly minted coins with no history, rather than coins that have a long and tainted history.

From these forms of differentiation, of quasi-uniqueness, then, the two problems merged. The problem of copying of digital assets could be solved with blockchain technology if the ledger were capable of recording tokens that had unique characteristics. A unique copy of an MP3, or a unique copy of anything else, for that matter, could be represented by an entry in a database, secured by cryptography and game theory against third party interference and with no need for an intermediary. Of course, it would be inefficient to create an entire blockchain for each type of unique digital asset needed (one for comic books, one for digital art, one for items in a virtual game, one for collectors' editions of albums, and so on). Among other things, such a design would mean that each blockchain would be less secure, since less work—processor cycles—would be dedicated to securing the database. However, a blockchain is programmable because it remembers state, which means blockchains can themselves serve as the foundation for software that runs on the distributed database. And that software can be other databases, much like Google Drive runs on Google's own databases—virtual machines—that in turn run on hardware machines. In the same way, a database listing unique tokens, virtual deeds that are as different from one another as Park Place and Mediterranean Avenue are in Monopoly, can be programmed to use the original blockchain, usually Ethereum, as a foundation, using a protocol called ERC-20 (an earlier version) or ERC-721 (and more protocols are forthcoming as community members proposed different formats).

Non-fungible tokens are database entries, written to a smart contract, which is a database itself, along with a number of rules for moving and identifying tokens. The smart contract lists the number of tokens issued, and the accounts to whom those tokens are assigned as entries in the contract, and sometimes rules for transfer or other features of the pool of tokens. The contract can specify certain rules on transfer—like remitting a percentage of the value of a sale back to the token's original creator—or other special rules that are not at all apparent to the purchaser without delving into the specifics of the smart contract.

Non-fungible tokens often do not represent value merely by themselves. A bitcoin is valuable purely because the entry in the bitcoin blockchain is valuable—humans want them and are willing to trade value for them. Nothing more is required. But many NFTs represent unique assets, or seek to make assets unique by metaphorically stapling a unique entry in a smart contract, a token, to an otherwise easily copyable intellectual property asset. Take, for example, Top Shot, a licensed digital collectible marketplace, which is run by the NBA. People purchase “Moments,” a.jpg of a few seconds worth of dramatic gameplay. What makes the “Moment” unique—and thus worthy of collection (since anyone who had access to the game could screen grab and make a.jpg of the same shot, steal, or free-throw), is that the “Moment” is associated with an NFT, a cryptographically unique token, an entry in a smart contract stating that buyer B owns Moment 1. The intellectual property license and the personal property interest in the token are in many cases only loosely associated. Usually the token contains a database entry of a url pointing to the .jpg, which is hosted on servers. Or, perhaps, the token contains a hash of the entire short film segment, a number generated by running all of the pixels of the.jpg through a mathematical function that creates a unique math string of limited length. That string, embedded in the list of features that make the token unique and recorded by the smart contract, proves that the token is associated precisely with—and only with—the original .jpg. It's a virtual staple, linking intellectual property to digital property, much like a link links one web page with another.

This look under the NFT hood cues up the questions raised in the following sub-parts, in which we analyze how the various questions of information theory are addressed, ignored, or actively swept under the rug. What role does virtual “thing-ness” play? How good of a simulacrum are NFT creators and buyers working with?

In each of the following sub-parts, the arguments track a general trend. Intuitions about property, combined with the informational elements of thing-ness, combine to provide an informational backdrop, traditions about what an owner may do with scarce resources. To the extent that a property scheme draws on established traditions, it conserves information costs. For example: It would be an odd property system that would not allow an owner to make use of their property. Some use restrictions therefore catch owners by surprise—particularly those that are the result of private dealmaking (a negative easement, for example) rather than public deliberation (i.e., zoning). To the extent that the bundle of rights and technological features meet buyers' expectations, the law of property for virtual things will make purchasing and using NFTs easier simply by meeting expectations. Yet, as we will see below, the artificial thing-ness of NFTs works out in some different ways as compared to physical thing-ness, and the legal regime surrounding intellectual property has so long ruled the digital asset space that the intuitions of personal property no longer obtain.

## Excludability, scarcity, and uniqueness in NFTs

Excludability, scarcity, and uniqueness are the strong suits of NFT frameworks. The tokens are mathematically and provably unique, the cryptography used in the blockchain structure ensures that each token is what it appears to be, and the combination of proof systems (proof of work being still the lead example) with game theory ensures that transfers do not result in double spending.

Yet there are components to the excludability and rivalrousness discussion that are not entirely resolved through NFTs. Virtual thing-ness may have successfully invoked the human urge to collect, but it has not resolved the human urge to copy.^38^ Take, for example, the celebrated $69 million NFT minted based on several years' worth of daily artwork by the artist Beeple.^39^ Would you like to see what it looked like? A simple Google search will work. Would you like to have your own copy? Right-clicking and saving the file will work. The same is true for depictions of the Mona Lisa: take a picture with your smartphone and you have your very own, and yet there are important differences. NFTs do not—directly—solve the copy protection problem. If a book is distributed with an NFT for each copy of the book, pirates who do not wish to pay for the book may still download it quite successfully. Some technological solutions to that problem may exist such as license servers.^40^

There are knock-on effects as well. Excludability bears on the eponymous Hohfeldian right to exclude, commonly theorized to be the most important of the property bundle of sticks. If I cannot keep someone else from accessing or using an asset, it is not functionally excludable or rival: forced sharing precludes excludability. One common way of expressing the right to destroy is as an extreme example of the right to exclude—the owner excludes everyone from the asset, including herself.^41^ Here, the nature of an NFT causes a split in the ability to exercise strong rights to exclude, including the right to destroy. A cryptographic token is of course easy to destroy in a manner of speaking. A transfer of a token to an account that does not exist means that the token can never be transferred again. This is termed “burning” the token, and is an integral part of some blockchains, which need a way of “destroying” database entries that are permanently and indelibly written to a public database.

But the dual nature of many NFTs—half token, half intellectual property—make exclusion or its ultimate extension—destruction—more complex. Consider an art-linked NFT. The token can be burned, but the intellectual property linked to it almost certainly won't be. Most tokens merely link to the IP file, which is hosted generally on some third-party server.^42^ A hash of the NFT and its URL link the token to the IP, but burning the token would in the overwhelming majority of cases not serve to destroy the intellectual property component of the NFT.

Again, there are workarounds, and again one might reasonably ask why a user would expect to be able to destroy something she owns. To the second question, destruction is a powerful statement—ask Banksy^43^—and anyway, the point is only that NFTs do not permit exclusion from an owned resource, merely a claim of association or affiliation by the owner. And to the first point, were NFT creators to decide to create versions of NFTs that act more like physical personal property, to give them the “thing-ness” characteristic of exclusion or destructibility, they could do so. Imagine an art NFT that was itself encrypted, and must be decrypted by the owner in order to view or use. If the decryption key to encrypted art were burned, and if there were no other decrypted copies of the file, then the piece would be effectively destroyed.

Although we have been discussing destruction here in order to explore how NFTs work differently than physical things for purposes of the Hohfeldian exclusion right, the limits on NFT exclusion apply in much less extreme cases. Consider, for example, that there is nothing that limits an IP rightsholder from minting another NFT connected to the same artwork, or indeed minting many such.^44^ The effect would be as though a baseball card company suddenly printed many more of a rare series, leading collectors to either be forced to differentiate between first and later created cards, or watch the value of the original plummet with each additional piece made available. Perhaps the age of a token will stand in for the collector's avid desire to own a black-border Black Lotus Magic the Gathering card, a phenomenon by which thing-ness and time combine to generate scarcity and value. But that will be a social process, one in which certain serial numbers or minting dates will grant and hold value for the NFT. It remains to be seen whether the communities that generate social value of affiliation will choose to map the technological features to social status. If they do not, then even the NFT owner's claim to exclusive affiliation with a piece of art or other tokenized asset will be fragile and difficult to value.

## Numerus clausus, fragmentation, and search costs

Consider the impact of the current technological and legal landscape on search costs and the informational costs posited by Smith, Merrill, and others. Simply put, what does an investor or buyer of NFTs get when they buy? What are the costs of finding out? The simple answer is that nobody has the vaguest idea because of several distinct features of the NTFs themselves (in particular the tension between intellectual property licenses and personal property interests), the movement toward fractionalization of interests in NFTs, and coded governance rules in the smartcontracts that govern both NFTs and govern fractional interests. This section examines each problem in turn.

### Numerus clausus

The initial problem is that the number of property forms in NFTs is not limited. EC-20 and EC-721 each permit quite different characteristics to be assigned to NFTs. A purchaser of an NFT has very little idea of what she is receiving. It is as if each NFT were its own form of property, with not only its own physical or aesthetic features, but with its own legal characteristics.^45^ Some NFTs will kick back a portion of their sale price each time they are sold.^46^ Some have a built-in capacity to be frozen from further sale by their creator.^47^ And so on. Thus, to begin with, the differences between different forms of NFTs create and exacerbate search costs for potential purchasers.

There is a stark informational line for physical things between physical attributes, which are visible, and legal attributes—information attached by law or documentation to the thing—which are not. Those are the ones that raise search costs. The line is fuzzier for NFTs. All of an NFT's features are informational in some sense or other. Some are public facing, for example, a gif or jpeg that constitutes the NFT in popular understanding. Those elements are highly visible. My drawing of a cat will not demand the same price as Beeple's *Everydays: the First 5,000 Days*, and buyers will easily be able to respond to the difference in those aesthetic characteristics because the picture is out in front, so to speak. But other characteristics of the NFT will require more effort to uncover, for example, whether the NFT imposes a royalty payment or percentage kickback on resale. These hidden informational costs are the exact problem that Smith and Merrill seek to address with the numerus clausus.^48^

The legal rights attached to an NFT token are unclear at best (particularly as regards intellectual property rights). Some of the features of the NFT are included in the smart contract that generated the token, not in the token / IP bundle that makes up the NFT. Some features are not immediately apparent, and there is no easy way to determine the characteristics of an NFT from the perspective of a surface-level buyer, someone who is simply bidding on a piece of online art, for example. There is no standard form for an NFT, nor a standard set of rights that attach to purchasing a token, either technological or legal. In short, NFTs impose significantly higher search costs on the buyer than would the current set of legal and informational features attached to a physical piece of personal property.^49^ To be clear, with standardization, these search costs may fall, and if a standard set of features and rights emerges from the current morass of different forms, the market may converge on a favored form. But it will take many rounds of standardization, and certainly a standard set of assumptions set by law to create a virtual *numerus clausus*. Until then, the costs of ascertaining exactly what one is buying when one buys an NFT remain quite high.

### Fragmentation

Rights in NFTs are deeply fragmented. A buyer of an NFT does not have clear rights to use, modify, destroy, or even sell what she bought. After all, most NFTs purport to carry some interest in intellectual property alongside the NFT, the intellectual property is an important part of the NFT valuation (consider again the $69-million-dollar digital-art NFT sold by Beeple), and yet the licenses for such art are usually deeply restrictive, imposing limits on the buyer that no collector of personal property would stand.^50^

IP rights layered with personal property rights pose a traditional fragmentation problem. NFTs have two other layered problems of fragmentation. The first has to do with fractional ownership of the NFT. Imagine owning an expensive collectible, the equivalent of an internet Mona Lisa. The promise of NFT fractionalization is based on infinite divisibility. Physical things have a fuzzy lower bound to fractionalization: ownership of a small part of a physical object is at some point mere ownership of a monetary interest, rather than any interest in the object itself. Owning half of a hammer, or entering into a co-tenancy ownership arrangement for a farm makes some sense: there is not only the monetary interest in the half part of the object or real estate, but that ownership interest also carries use of the thing. At some point, however, ownership interests become too small to convey any practical non-financial use of the thing. Consider a house with 10,000 owners: the co-tenancy cannot possibly be useful, it must purely be financial. And given that the practical ability to use a thing disappears as large number of fractional owners enter play, the value of the asset itself declines by the amount of its use. It cannot be used, merely traded, and that is a loss.

These issues have complex relationships to the new class of digital things. For example, were the IP licenses so worded, each owner of the digital thing might be permitted to use the licensed work: each fractional owner of the internet Mona Lisa could put her likeness on their social media page, or what have you. Social media use is a bit of a specious example, but recall that the point of ownership is most often to associate oneself with the good in some unique fashion. And there lies the rub. Leveraging the non-physicality of NFTs to turn fractional interests into full use rights—everyone can have and be associated with their own copy of Beeple's Everydays: the First 5,000 Days, and a piece of internet history as long as they own a fraction of the NFT—dilutes exactly the uniqueness that NFTs created. Fractionalized ownership either undoes the careful work of creating excludability or rivalrousness (everyone can own a minute fraction of an NFT that conveys full use rights and association) or it does not (fractionalized ownership rights merely convey a financial interest in the NFT). Neither outcome works.

More, assuming the latter and more likely outcome, that NFTs retain their excludable characteristics despite everyone being able to own a miniscule piece of them, fractionalized ownership will raise search and related information transaction costs. Imagine the shift from the problems listed by Smith, Merrill, and others—a piece of property burdened with cross-cutting property interests, freezing the asset in the stream of commerce—and multiply the problem many times over. The first problem is the sheer number of owners. A house co-owned by another person, or burdened with a single easement is one matter. An asset burdened by tens of thousands of crosscutting rights is another matter entirely.

NFT creators and sellers are not unaware of the problem. Companies like Fractional seek to provide not only a means of fractionalizing tokens by minting more tokens to represent fractional interests in the first token (and there is nothing to stop one from fractionalizing the fractionalized tokens, it's turtles all the way down), but also to provide governance rules for fractional interest purchasers. After all, if one is putting a few dollars in to invest into ownership of a very expensive NFT, the primary interest is financial, and the fractional interest holders will very much want to be heard on whether, when, and under what terms the NFT would be sold. These governance rules, though, have some very strange characteristics themselves. First, they are only internal to any one fractionalization scheme. So, say that a co-owner of interests in a token decided to fractionalize her 50% interest in an NFT using Fractional. Assuming that Fractional is serious about developing governance rules, particularly as relate to sales of the interests, that half-interest could be governed by the Fractional rules. Imagine another (hypothetical) company, Part.ly, that has the same business model of Fractional, but slightly different governance rules. Part.ly fractions would govern the other fractionalized interests. In principle, there is no limit to the different governance regimes that could rule internal determinations of what is to be done with a valuable NFT.

### Modularity

The last function of thing-ness identified by property theorists following the informational turn is that thing-ness encapsulates complexity. Consider a printer cartridge: easily swappable, but if opened, the module contains considerable complexity. Note that we call a cartridge a cartridge without decomposing it into ink and ribbon and so on: the thing is the physical boundary of plastic that binds all of the components together, and makes it easily modular with the rest of the system.

The question is to what degree and in what context do the efforts at virtual thing-ness encapsulate complexity and permit modularity? Interoperability and modularity in blockchain applications work in a number of ways. Consider Ethereum. The blockchain both serves as currency for running programs on the blockchain's virtual machine, and as the virtual machine itself. NFTs are often purchased with ether, and the smart contracts that determine who owns which NFT are often themselves programs riding on the Ethereum blockchain. Tokens that are swappable for ether therefore have both the modularity of a single blockchain and the exchangeability of a common currency.

However, modularity in tokens raises new questions of complexity. NFTs are of course simulated things, not physical things. Portability is a real issue. Whereas a hammer purchased in a hardware store can be taken to any job site, tokens are not free of the nested context in which they are generated. A token is, after all, only an entry in a smart contract ledger pointing to a given account as the owner. The token is not exportable outside of the list that gives it meaning; it is as if the hammer can never be truly taken out of the store.

Similarly, NFTs are not fully portable outside of the user-facing context in which many are situated. Consider an NFT of a card in a collectible card trading game—Gods Unchained, for example. The card only has meaning when played within the playing environment created by the minter of the card. The graphics only display, the card attributes only take effect, the game only goes on within the environment provided by the card creator.

There are some attempts to create modularity and portability for NFTs. The drafters of the Nifty License that governed Cryptokitties, an early breaking NFT application, opened the license rights in the IP (the pictures of the cryptokitties themselves) to permit cryptokitties to be used in other contexts. Thus, for example, if a third party created a game in which cryptokitties could race one another, the IP license would contain a limited carveout for purposes of portability.

Because NFTs are informational objects, they are more dependent on information environments (wallets for tokens, environments for game elements, virtual museums for art collections and the like) to give them life. Pure art NFTs are somewhat more portable than other instances, because they can (one supposes, although the licenses generally do not confirm) display them in an electronic environment of the owner's choosing, from Twitter to museums in Flatland. What is clear, however, is that the element of physicality that makes a hammer fully portable to new environments—physics is in this sense a set of mutually operable rules that work regardless of environment—works out differently in the NFT context. Portability and interoperability are a problem because of these external dependencies on things beyond the NFT itself. And the NFT may not encapsulate internal complexity well either. The token may not contain certain idiosyncrasies or features: they may be listed in the smart-contract that generated it. Thus, NFTs lack a surface, a natural thing-ness, that ensures that they operate as a unit, that they encapsulate all necessary elements for function.

## Conclusion

The creation of NFTs is an unabashed and long overdue attempt at reification, at turning information into objects by listing a feature set (for example, excludability) that mimics the characteristics of physical objects, with the goal of enabling and tapping the human desire to collect rare and unique objects. They have been a runaway success, in that the market for NFTs exploded, and a profound legal failure, in that the present meltdown reflects the legal feet of clay of the entire market. The virtual objects made during the NFT minting process, an amalgam of cryptographic database entries, intellectual property, and social value that attaches to the whole, do not increase owners' knowledge of what they have purchased, reduce search costs, or enable modularity in the way that property theorists of the informational turn have noted for physical property.

The above critique should in no way be taken as a lack of confidence about the future of NFTs: true digital uniqueness has long been a holy grail, and even without strong protections, gray markets in virtual property have thrived for decades. Rather, by understanding how attempts at thing-ness have not quite achieved their goals, we can see what is yet to be done. Intellectual property must take a backseat to personal property interests, so that buyers may use and display their purchases. Increased standardization in the forms of NFTs are necessary to lower search costs and increase buyers' understanding of what they have purchased. Personal property rights over NFTs are and must continue to be recognized by courts, to allow buyers to rely on their broad understanding of the set of things they may do with their property. And creators who wish to increase the value of their offerings will have to find ways to increase modularity and portability. Without these changes, NFTs will remain a real risk: buyers simply cannot know what they have bought, and they do not know what it means to own a piece of unique digital property; their intuitions will lead them astray, and they will be tripped up by hidden code and obscure legal doctrine.

## Data availability statement

The original contributions presented in the study are included in the article/supplementary material, further inquiries can be directed to the corresponding author.

## Author contributions

The author confirms being the sole contributor of this work and has approved it for publication.

## Conflict of interest

The author declares that the research was conducted in the absence of any commercial or financial relationships that could be construed as a potential conflict of interest.

## Publisher's note

All claims expressed in this article are solely those of the authors and do not necessarily represent those of their affiliated organizations, or those of the publisher, the editors and the reviewers. Any product that may be evaluated in this article, or claim that may be made by its manufacturer, is not guaranteed or endorsed by the publisher.

## Footnotes

^1^*See* Noonan (2009) (“Today, transacting electronically has become the norm rather than the exception. Nearly any type of contract can be drafted and executed electronically.”).

^2^*See* Fairfield (2009) (describing how End User License Agreements may present an issue of meaningful consent in contract).

^3^*See* Moringiello (2007) [hereinafter Moringiello, *False Categories*] (explaining the “tendency to place new intangible rights into the category of intellectual property in case law and scholarship”).

^4^*See* Fairfield (2017) [hereinafter Fairfield, Owned] (explaining how the RAM Copy Doctrine and DMCA 1201 contributed to the treatment of intangible (digital) property differently than its physical analog, under intellectual property laws).

^5^*See, e.g*., Stone, 2009.

^6^*See* MAI Sys. Corp. v. Peak Comput., Inc., (“Peak's loading of copyrighted software into RAM creates a “copy” of that software in violation of the Copyright Act.”).

^7^*Id*.

^8^*See* Moringiello, *False Categories, supra* note 3, at 148–50 (describing the conflict for intellectual property law to govern ownership of a domain name).

^9^Kremen v. Cohen (“Property is a broad concept that includes every intangible benefit and prerogative susceptible of possession or disposition.”) (internal quotes omitted).

^10^*See* Fairfield, Owned, supra note 4, at 45–48 (summarizing the intellectual property and online contract regime governing digital property rights).

^11^*See, e.g*., Robertson, 2021.

^12^*See* A&M Records, Inc. (explaining how Napster enables copyright infringement).

^13^*Id*.

^14^Fairfield, Owned, *supra* note 4.

^15^*See* Perzanowski and Schultz (2016).

^16^*See* 17 U.S.C.A. § 1201 (West) (“No person shall circumvent a technological measure that effectively controls access to a work protected under this title.”).

^17^*See* Fairfield (2015) [hereinafter Fairfield, *Bitproperty*].

^18^(Beer, 2021).

^19^*See generally* Merrill and Henry (2000) [hereinafter Merrill & Smith, *Numerus Clausus*].

^20^*See id*.; Smith (2012) [hereinafter Smith, *Property As the Law of Things*]; Merrill and Smith (2001).

^21^*See* Smith, *Property As the Law of Things, supra* note 20 at 1698 (“[The] principle that property forms come in a finite and closed menu.”). Cite numerus clausus article.

^22^*See id*. at 1694 n.8 (“It is worth noting that strategies for managing rights to use open-access resources tend also to rely on simple, easily known rules, which also economize on information costs.”).

^23^*See e.g., id*. at 1706 (“In rem rights are directed at a wide and indefinite audience of duty holders and other affected parties, who would incur high information costs in dealing with idiosyncratic property rights and would have to process more types of information than they would in the absence of the *numerus clausus*.”).

^24^*See* Merrill and Smith, *Numerus Clausus*, *supra* note 19 at 27 (explaining that sales of “time-shares” of a property can only be done through contract and no such interest in property can be transferred).

^25^*See*Latour (1993) (“Quasi-objects are in between and below the two poles [of nature and society], at the very place around which dualism and dialectics had turned endlessly without being able to come to terms with them. Quasi-objects are much more social, much more fabricated, much more collective than the “hard” parts of nature, but they are in no way the arbitrary receptacles of a full-fledged society.”).

^26^*See* Geertz (1973) ([M]an is an animal suspended in webs of significance he himself has spun.”).

^27^*See* Davidson (2008) (explaining the value of numerus clausus).

^28^*Id*.

^29^*See* Merrill and Smith, *Numerus Clausus*, *supra* note 19 at 40–42 (describing how property conceptualized in terms of the cost of maintaining and searching property ownership interests is useful).

^30^*See* id. (“Consider the rise of registers of interests in real property, that is, recording acts. This device lowers the costs of notice; it is an alternative method of lowering information costs.”).

^31^*See* Smith, *Property As the Law of Things, supra* note 20 at 1700–07 (2012) (analyzing “thing-ness” and the modular conception of “things” in property).

^32^*Id*.

^33^*Id*.

^34^*See* Fairfield, *Bitproperty, supra* note 17 at 839 (“Traditional property, a system designed through a long tradition of common law deliberation to govern interests in scarce and rival resources, did not seem at the time of the rise of the Internet to be immediately applicable to an environment in which many resources were neither scarce nor rival.”).

^35^*Id*.

^36^*See* Smith, *Property As the Law of Things, supra* note 20.

^37^*See* Fairfield (2021) [hereinafter Fairfield, *Tokenized*] (“The creator of the system has significant control over the [NFT] because they are able to ban or control access to the service or site in which the asset is used.”).

^38^A recent example of item duplication in the popular open-world game, Valheim, exemplifies how people still have an interest in “duping” virtual items whenever they find the chance. *See* Zimbler, 2021.

^39^*See* (Kastrenakes, 2021) (reporting on the record-breaking NFT sale).

^40^*See Software License Server* (“To keep track of the licenses and users, the license server uses a centralized computer software system that gives access tokens—also known as software license keys—that allow licensed software to run on a client's computer. No token—no access.”).

^41^Strahilevitz (2005) (“The right to destroy property is, after all, often an extreme exercise of some of the more widely recognized sticks in the bundle of rights. The right to destroy is an extreme version of the right to exclude; by destroying a vase, I permanently exclude third parties from using it.”).

^42^*See* Finzer (describing how NFT art is most often stored on a third-party server).

^43^*See* Reyburn, 2018.

^44^There is nothing stopping NBA TopShot from minting more versions of a high-selling GIF after a user purchases the original copy. *See* Terms of Use (2022) (explaining that the user owns the token and the company owns the IP).

^45^For an exploration of the different forms of NFTs, see Fairfield, *Tokenized, supra* note 38 at 44–48 (creating a taxonomy of NFT forms).

^46^*Id*.

^47^*Id*.

^48^*See* Merrill & Smith, *Numerus Clausus*, *supra* note 19 at 24–38 (discussing different costs that result from too many property forms).

^49^*Id*.

^50^*See Terms of Use (**2022**) (explaining that the user owns the token and the company owns the IP)*.
